# iRGD-modified memory-like NK cells exhibit potent responses to hepatocellular carcinoma

**DOI:** 10.1186/s12967-023-04024-7

**Published:** 2023-03-17

**Authors:** Yanbing Dong, Ying Huang, Zhe Zhang, Aoxing Chen, Lin Li, Manman Tian, Jie Shen, Jie Shao

**Affiliations:** 1 Comprehensive Cancer Centre of Drum Tower Hospital, Medical School of Nanjing University, Clinical Cancer Institute of Nanjing University, Nanjing, China; 2 Comprehensive Cancer Center, Nanjing Drum Tower Hospital Clinical College of Traditional Chinese and Western Medicine, Nanjing University of Traditional Chinese Medicine, Nanjing, China; 3 Department of Oncology, The Second People’s Hospital of Huai’an, Huai’an, China

**Keywords:** Memory-like NK cells, Hepatocellular carcinoma, iRGD

## Abstract

**Background:**

Cytokine-induced memory-like natural killer (CIML NK) cells have been found to possess potent antitumor responses and induce complete remissions in patients with leukemia. However, the poor infiltration of transferred NK cells is a major obstacle in developing adoptive cell immunotherapy for solid tumors. In our study, we explored the potential of using the tumor-penetrating peptide iRGD to deliver activated CIML NK cells deep into tumor tissues.

**Methods:**

After being briefly stimulated with interleukin-12 (IL-12), IL-15, and IL-18, CIML NK cells were assessed for their phenotype and function with flow cytometry. The penetrating and killing capability of iRGD-modified CIML NK cells in tumor spheroids was revealed by confocal microscopy. The anti-tumor efficacy of these modified CIML NK cells was tested in hepatocellular carcinoma (HCC) xenograft mouse models.

**Results:**

Treating NK cells with cytokines led to a substantial activation, which was evidenced by the upregulation of CD25 and CD137. After a resting period of six days, CIML NK cells were still able to display strong activation when targeting HepG2 and SK-Hep-1 HCC cell lines. Additionally, CIML NK cells produced increased amounts of cytokines (interferon-gamma and tumor necrosis factor alpha) and exhibited heightened cytotoxicity towards HCC cell lines. The iRGD modification enabled CIML NK cells to infiltrate multicellular spheroids (MCSs) and, consequently, to induce cytotoxicity against the target cancer cells. Moreover, the CIML NK cells modified with iRGD significantly decreased tumor growth in a HCC xenograft mouse model.

**Conclusion:**

Our findings demonstrate that CIML NK cells possess augmented potency and durability against HCC cell lines in vitro. Additionally, we have seen that the incorporation of iRGD to CIML NK cells facilitates enhanced infiltration and targeted destruction of MCSs. Moreover, the application of iRGD-modified CIML NK cells reveal remarkable anti-tumor efficacy against HCC in vivo.

**Supplementary Information:**

The online version contains supplementary material available at 10.1186/s12967-023-04024-7.

## Background

Primary liver cancer is one of the most commonly diagnosed forms of cancer worldwide in 2020, with approximately 906,000 new cases and 830,000 deaths [1]. The majority of these cases are hepatocellular carcinoma (HCC), with the highest rates recorded in Eastern Asia (Age-standardised incidence rate: 14.8) [2]. When diagnosed early, the primary treatment for HCC is surgical resection; however, many patients are diagnosed at advanced stages, making systemic therapies the only option. Despite being potentially curable with hepatectomy, nearly 70% of patients relapse within five years [3]. Recently, immune checkpoint inhibitors have revolutionized the management of HCC, yet the factors associated with adverse clinical reactions and molecular pathways to drug resistance remain largely unknown [4]. Consequently, there is an urgent need for effective adjuvant therapies to reduce the recurrence for HCC post-curative treatments.

Immunotherapy has recently become a significant component of cancer treatment, with adoptive cell therapy (ACT) being one of its most important forms [5]. ACT involves the infusion of tumor-infiltrating lymphocytes or peripheral blood-derived immune cells, such as T lymphocytes (T cells) and natural killer cells (NK cells), into patients. NK cells are generally known to be effective in the innate defense against tumor growth and metastases, with a 73% response rate being observed in clinical trials involving hematological malignancies [6]. However, the response rate for NK cells adoptive transfer immunotherapy is much lower when it comes to solid tumors such as colorectal cancer, non-small cell lung cancer, melanoma, and hepatocellular carcinoma. This poses a considerable challenge for NK cell-based therapy to be used effectively against solid tumors.

It has been established that there is a positive correlation between the density of NK cells and the prognosis of HCC patients, indicating a critical role of NK cells in controlling tumor growth [7]. Cytokine-induced memory-like (CIML) NK cells, which differentiate after being activated with interleukin-12 (IL-12), IL-15, and IL-18, have been observed to possess enhanced anti-tumor responses. Preclinical and clinical studies suggest that memory NK cell activities could be beneficial in tumor settings and may contribute to relapse prevention in the context of hematopoietic malignancies[8, 9]. A key feature of memory-like NK cells is their extended lifespan and the ability to generate persistent responses. Despite this, CIML NK cells have not yet achieved significant clinical success in the treatment of solid tumors, unlike myeloid malignancies. This is due to factors that may impede the homing and penetration of NK cells into the deeper areas of tumor masses, such as hypoxia, lack of visible NK cell receptors, and desmoplastic stroma in the solid tumor setting[10–13].

The iRGD peptide (sequence: CRGDKGPDC) mediates the permeability of tumor blood vessels, resulting in internalization and penetration into tumor tissues, thereby improving diagnostic accuracy and therapeutic efficacy [14–16]. Our team has also reported that iRGD can enhance the infiltration of transferred T cells into tumors [17]. Specifically, T cells that were coated with iRGD connected to 1,2-distearoyl-sn-glycero-3-phospho-ethanolamine-polyethylene glycol (DSPE-PEG) demonstrated a significantly increased ability to penetrate deep into tumor tissues. Furthermore, combining iRGD modification with PD-1 knockout lymphocytes has been shown to be highly effective in multiple xenograft mouse models.

In this research, we posited that combining two strategies to improve NK cell-mediated anti-HCC responses, namely memory-like differentiation and iRGD-modification, would be a feasible and efficient immunotherapeutic approach for HCC. Our results showed that IL-12, IL-15, and IL-18-activated NK cells had memory-like properties, which resulted in an increased production of IFN-γ and TNF-α upon restimulation with tumor cells and heightened cytotoxicity when compared to IL-15-maintained NK cells. Furthermore, we also established that CIML NK cells modified with iRGD had enhanced penetration capacity and killing capacity in MCSs and a HCC xenograft tumor model.

## Materials and methods

### Cell lines

The cell lines HepG2, SK-Hep-1, HUVEC and iRGD receptor-positive HGC27 were purchased from the Cell Bank of the Shanghai Institute of Biochemistry and Cell Biology. The cells were cultured in RPMI-1640 medium which was enriched with 10% fetal calf serum, 100 U/mL penicillin and 100 µg/mL streptomycin at 37 °C and 5% CO2. The cell identities were authenticated by phenotype or genotype and only Mycoplasma-free cells were employed. Human dendritic cells were generated as previously described [18]. In brief, dendritic cells (DCs) were generated from monocytes enriched by adherence for 2 h, and then cultured in AIM-V medium containing human GM-CSF (500 U/ml, Peprotech) and IL-4 (500 U/mL, Peprotech) for 5 days. To obtain mature DCs, fresh complete medium containing LPS (10 ng /mL, Sigma) and IFN-γ (500 U/mL, Peprotech) was added to the culture on day 5. The culture was continued for an additional 24 h.

### NK cells purification and generation of CIML NK cells

PBMCs were collected from patients with informed consent and used to isolate primary NK cells via CD56 positive selection (Miltenyi Biotec, Germany). For cytokine induced-memory-like and control NK cells, NK cells were cultured at 3–5 × 10^6^ cells/mL and pre-activated for 16 h (on day 1) with a mixture of 10 ng/mL IL-12 (Amoytop, China), 50 ng/mL IL-15 (Amoytop, China), and 50 ng/mL IL-18 (Amoytop, China) or control conditions (IL-15, 3 ng/mL). After 16 h, the NK cell preparations were washed three times with PBS (Hyclone) to remove cytokines and placed in a complete AIM-V medium containing 10% fetal bovine serum (Gibico, USA) supplemented with IL-15 (3 ng/mL) to support survival. The medium was refreshed every 2 or 3 days, with additional IL-15 supplementation [11, 12].

### Flow cytometry analysis

A Cytoflex flow cytometer (Beckman Coulter, USA) was utilized to perform fluorescent expression analysis. For phenotypic NK cell characterization, the following antibody clones were employed: anti-CD16-PE (3G8, BD Biosciences, USA), anti-CD56-BB700(B159, BD Biosciences, USA), anti-CD56-APC (B159, BD Biosciences, USA), anti-CD3-FITC (HIT3a, BD Biosciences, USA), anti-KIR-APC (DX9, BD Biosciences, USA), anti-NKp30-APC-R700 (P30-15, BD Biosciences, USA), anti-NKG2A-BV650 (CD159a, BD Biosciences, USA). The frequency of cells that fell within the CD56^+^ gate was used to determine the cells expressing each antigen. Flow cytometry analysis of anti- CD25-APC (2A3, BD Biosciences, USA), and anti- CD137-PE (4B4-1, BD Biosciences, USA) was employed to assess CIML NK cells activated by cytokines. Anti-CD107a-PE (H4A3, BD Biosciences, USA) and anti-GranzymeB-PE (GB11, eBioscience, USA) were employed to assess CIML NK cells cytotoxicity when co-culture with HCC cells. Restimulation assays were performed by incubating NK cell preparations with target cells (K562, HepG2, HGC27, or SK-Hep-1 cells) at an effector-to-target ratio of 2:1 for 6 h or 24 h in complete medium (supplemented with 3 ng/mL IL-15) one week after initial stimulation. Cells were washed, and their expression of different markes was analyzed through antibody staining and flow cytometry, as previously described. Expression of iRGD receptors on HepG2, monocyte, DC and HUVEC was measured by staining cells with anti-αvβ3-FITC (LM6090, EMD Millipore), anti-αvβ5-FITC (P1F6, EMD Millipore), or anti-NRP-1-PE (AD5-17F6, Miltenyi Biotec GmbH). Monocytes were gated with anti-CD14-APC (M5E2, BD Biosciences, USA) and DCs were gated with anti-CD11c-APC (B-ly6, BD Biosciences, USA). Prior to analysis, all samples were suspended in flow cytometry staining buffer (FACS) buffer and stained with the indicated antibodies for 30 min at 4 °C in the dark, followed by two washes and resuspension in FACS buffer.

### Intracellular cytokine staining

Sorted CD56^+^ NK cells were pre-activated with IL-12, IL-15, and IL-18 for a brief period. After a 7-day rest period, ICS was conducted and 1 × 10^6^ CIML NK cells were re-stimulated with HepG2 cells at the designated effector to target cell ratios. Subsequently, GolgiStop (BD Biosciences) was administered to the stimulated NK cells in accordance with the manufacturer's instructions for 8 h. Following treatment, the NK cells were stained with a fixable live/dead stain (FVS780) and anti-CD56-BB700 for 30 min at room temperature. Cells were then fixed and permeabilized using the Fixation/Permeabilization Solution Kit (BD Biosciences). Intracellular cytokines were stained with anti-IFN-γ-PE (B27, BD Biosciences) and anti-TNF-α-APC (MAb11, BD Biosciences) for 1 h at 4 °C. After washing with permeabilization buffer, the cells were fixed with 1% paraformaldehyde solution (Sigma-Aldrich) and analyzed using a CytoFLEX.

### CD107a and granzyme B assays

For CD107a and granzyme B assays, sorted NK cells were pre-activated with IL-12, IL-15, and IL-18 or a control condition for 16 h before being differentiated into memory-like or control NK cells over a 7-day period. Following re-stimulation with either HepG2 or SK-Hep-1 cells at the designated effector to target cell ratio of 2:1, CD107a-PE antibody was added for the first hour, followed by GolgiPlug (BD Biosciences) for a further five hours. The cells were then washed, stained with a fixable live/dead stain (FVS780) and surface antibodies for 30 min at room temperature before being analyzed using a CytoFLEX. For granzyme B assays, NK cells re-stimulation with target cells for the first hour, cells were cultured with protein transport inhibitor GolgiPlug (BD Biosciences) for a further five hours. Following treatment, samples were fixed, permeabilized (Fixation/Permeabilization Solution Kit, BD Biosciences), and stained with anti-Granzyme B-PE (GB11, eBioscience, USA). After washing with permeabilization buffer, the cells were fixed with 1% paraformaldehyde solution (Sigma–Aldrich) and analyzed using a CytoFLEX.

### Cytometric bead array analysis of cytokines

For tumor cells restimulation, K562, HepG2 and SK-Hep-1 cells were added on day 7 to the respective NK cell preparation (E: T ratio of 2:1). After 6 h, the concentrations of cytokines in the culture supernatants were quantified using a cytometric bead array in accordance with the instructions provided by the manufacturer (BD Biosciences). The Human IFN-γ Flex Set (Bead B8) (BD Biosciences) was used to detect single-cytokine IFN-γ. The samples were analyzed by a Cytoflex flow cytometer and the data were analyzed with the help of FCAP version 3.0 array software (Soft Flow).

### Cytotoxicity assay

The cytotoxicity of CIML NK cells was evaluated in vitro against HCC cell lines (HepG2 and SK-Hep-1) on day 7 using a carboxy fluorescein succinimidyl amino ester and propidium iodide (CFSE/PI) assay. Initially, target tumor cells were labeled with 4 μM CFSE in a 37 °C incubator with 5% CO2 for 10 min. Following labeling, the cells were washed with PBS and then seeded into 48-well plates. The CFSE-labeled target tumor cells were then incubated with NK cells at various effector-to-target ratios at 37 °C and 5% CO2 for 6 h. Wash the cells with PBS(Hyclone) (5 min, 300 × g, RT) and resuspend the pellet in 100 μL PBS (Hyclone). To assess the ratio of cell death, PI (Sigma) was added. Finally, the samples were examined via flow cytometry.

### Synthesis of DSPE-PEG-iRGD

As previously described [17], DSPE-PEG-Mal (Laysan Bio, Inc, USA) and C-iRGD (ZPC, china) were mixed at a 1:1 molar ratio in Hepes buffer (pH = 6.5) and allowed to react at room temperature for 48 h under a nitrogen atmosphere. The reaction mixture was then dialyzed in deionized water for 48 h to remove any free iRGD, and the resulting solution was lyophilized and analyzed using MALDI-TOF MS and ^1^H NMR spectroscopy.

### The effect of CIML NK cells modified with DSPE -PEG -iRGD on multicellular spheroids (MCSs)

HGC27 cells were seeded in a 96-well plate (round bottom, ultra-low attachment surface, Corning, USA) containing RPMI1640 supplemented with 10% FBS (7500 cells/well). After attachment, half of the medium was replaced with RPMI1640 supplemented with 10% FBS. Daily observation under a light microscope was conducted to determine the formation of 'spherical' spheroids, and their diameters were measured using ImageJ software. When the spheroids had reached a size of around 500 µm, they were chosen for further research. In order to evaluate penetration, CIML NK cells and c-NK cells were cultivated for 7 days and then labelled with CFSE before being modified with DSPE-PEG-iRGD (20 µg modification reagent for every 1 × 10^6^ cells). These modified NK cells were then added to the multicellular spheroids (MCSs) at an effector-to-target (E:T) ratio of 5:1 which was calculated on the initial number of spheroids inoculated. After 6 h of incubation at 37 °C, the spheroids were washed for the removal of free NK cells, fixed in 4% paraformaldehyde, and imaged using a ZEN 710 confocal microscope (Zeiss, Jena, Germany). Images were acquired at the midheight of the spheroids and surface plots were generated using ImageJ software. For killing assay, NK cell preparations were modified with DSPE-PEG-iRGD and co-cultured with MCSs. After incubation, MCSs were treated with Calcein AM and Ethidium homodimer III (EthD-III) solutions (Viability/Cytotoxicity Assay Kit, biotium, USA) as per the manufacturer's instructions. The MCTS were then washed with PBS twice and placed in confocal dishes (In Vitro Scientific, Austria). The excitation wave-lengths were 494 nm for Calcein AM, 532 nm for EthD- III and the fluorescence signals were collected in 517 nm for Calcein AM and 625 nm for EthD-III. The images were then processed using ImageJ software.

### Xenograft mouse models

The Ethics Committee of Drum Tower Hospital (Nanjing, China) approved all experiments in this study. All animal procedures were conducted in accordance with the guidelines set by the Animal Care Committee at Drum Tower Hospital. The investigators were not blind to the animal studies. Every effort was made to decrease the number of animals used and to reduce their suffering. The mice were allocated randomly based on age and weight. For the subcutaneous tumor model, 6–8 weeks old male BALB/c nude mice were injected subcutaneously in the right axilla with HepG2 cells (5 × 10^6^ suspended in 100 μl PBS).

### In vivo near-infrared florescence imaging

To investigate the tumor targeting efficiency of CIML NK-iRGD in tumor-bearing mice, 10^7^ CIML NK cells stained with near-infrared fluorescent probe DiR (Bridgen, China) were injected intravenously (HepG2 subcutaneous tumor model). At different time intervals, the mice were anesthetized and scanned using a CRi MaestroTM Automated In Vivo Imaging System (C.R. INTERNATIONAL INC, USA). We also created a single-cell suspension of tumors after 24h that had been treated with CIML NK or CIML NK-iRGD and evaluated the infiltration of adoptive transferred NK cells by flow cytometry.

### In vivo antitumor efficacy

To create a subcutaneous tumor model, 6–8 weeks old male BALB/c nude mice were injected subcutaneously in the right axilla with HepG2 cells suspended in 100 μl PBS. Two weeks post tumor induction, the mice were treated with intravenous injection of 0.1 ml PBS, P–C-NK, P–C-NK-iRGD, P-CIML NK, or P-CIML NK-iRGD every seven days for a total of two times. Additionally, 50,000 U of human recombinant IL-2 was administered intraperitoneally every other day after cell transfer. At the time of adoptive cell transfer, the mice were weighed and checked for tumor development. The volume of the tumor was estimated by assuming an ellipsoid shape and calculated as length × width2 × 0.5. Lastly, major organs were collected, fixed in 4% paraformaldehyde, sectioned, and stained with H&E in order to assess safety. After retro-orbital blood was taken, Balb/c-nude mice with HepG2 tumors were put down, and surgical procedures were conducted to obtain subcutaneous tumor tissue and organs (heart, liver, spleen, lung, and kidney). These tissues were then fixed in 4% paraformaldehyde, sectioned, and stained with H&E to assess safety. Furthermore, mice blood samplings were analyzed for serum biochemical tests such as blood urea nitrogen (BUN), creatinine (Cr), alanine transaminase (ALT), aspartate transaminase (AST).

### Statistical analysis

GraphPad Prism v.9.0 was used for graphical representation and statistical analysis with all statistical comparisons indicated in the figure legends. Data are represented showing mean ± standard error of the mean (s.e.m.). All comparisons used a two-sided an of 0.05 for significance testing.

## Results

### Phenotypic characterization of CIML NK cells

**Fig. 1:**
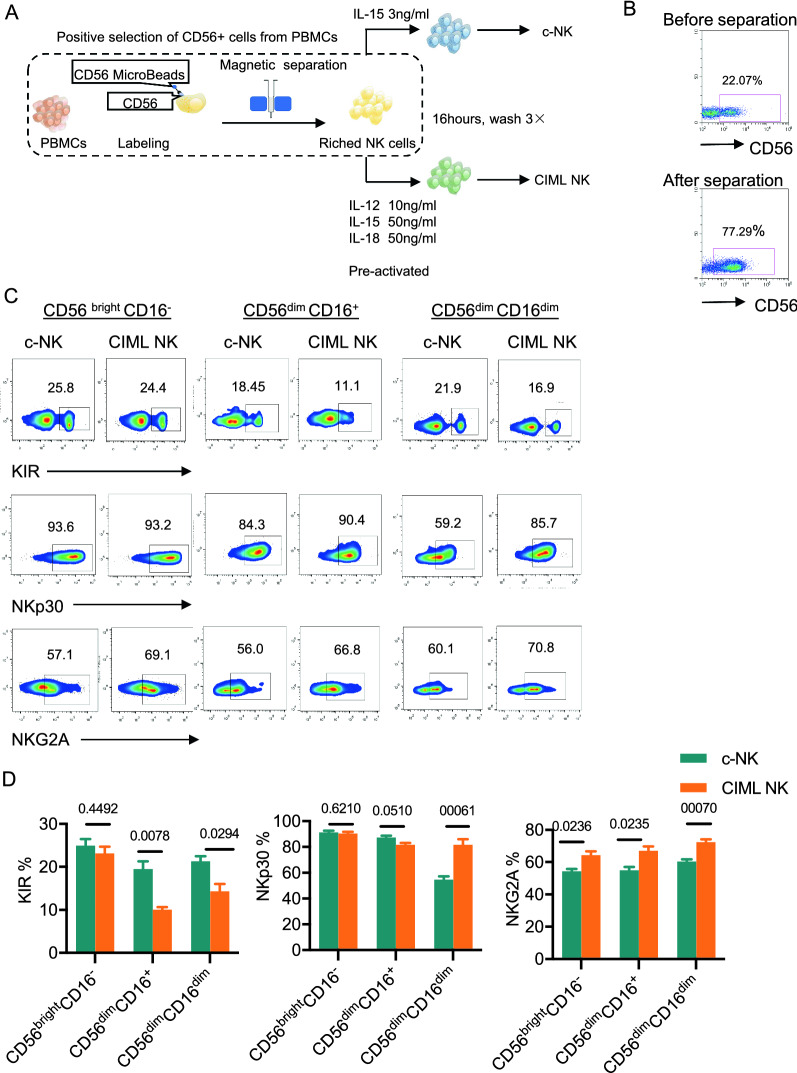
Priming NK cells with IL-12, IL-15, and IL-18 prompts trained memory-like phenotypic alterations among different NK subpopulations. **A** Schema of isolating NK cells from peripheral blood mononuclear cells and priming them with IL-12, IL-15, and IL-18 or low concentrations of IL-15 for 16 h, washing and then resting in a low concentration of IL-15 to allow for differentiation in vitro. **B** Representative flow plots showing the frequency of CD56 positive cells before and after positive separation. **C** Representative flow plots showing c-NK cells and CIML NK cells. Insert values are the percent positive of indicated markers for c-NK cells and CIML NK cells that fall within the indicated CD56 and CD16 gate. **D** The bar graphs show a comparison of c-NK and CIML NK cells from a representative individual on 3 NK cell phenotypic markers of different NK cell subsets. All values shown are mean ± s.e.m. of triplicate or duplicate measurements and have been repeated 3 times with similar results

PBMCs were separated by means of centrifugation on a Ficoll density gradient and then suspended in AIM-V medium (Gibico, USA). NK cells were purified using CD56 positive selection magnetic bead (Miltenyi Biotec, Germany). Sorted NK cells were preactivated with IL-12, IL-15, and IL-18 or low-dose IL-15 as a control (Fig. 1A, 1B). After 16 h, the phenotypic characterization of CIML NK cells was assessed using flow cytometry. NK cells are typically divided into two subsets based on the surface expression of CD56. CD56^bright^ NK cells produce large amounts of cytokines such as interferon-gamma (IFN-γ), which can regulate other immune cells or directly affect tumor cell viability, while CD56^dim^ NK cells are involved in direct cell-mediated cytotoxicity [19]. NK cells execute their functions through germline-encoded receptors which transmit activating or inhibitory signals [20]. Upon examining the surface expression of KIR, NKP30, and NKG2A on IL-12, IL-15, and IL-18-primed NK cells, it was observed that there were variations in the expression levels of the NK subgroups. Figure 1C and 1D demonstrated that the KIR expression on CIML NK cells was lower than the control group in the CD56^dim^ CD16^+^population (10.03 ± 0.6064% vs 19.47 ± 1.812% for CIML NK versus c-NK in CD56^dim^CD16^+^, and 14.7 ± 1.739% vs 21.3 ± 1.193% for CIML NK versus c-NK in CD56^dim^CD16^dim^; P = 0.0078 and P = 0.0294, respectively). Analysis revealed no significant difference in the proportion of c-NK cells and CIML NK cells expressing NKp30 in the CD56^bright^CD16^−^ population (91.33 ± 1.202% vs. 90.33 ± 1.453%, P = 0.6240) or in CD56^dim^CD16^+^ population (87.33 ± 1.453% vs. 81.67 ± 1.453%, P = 0.0510). The pre-activation strategy was found to increase NKG2A expression in CIML NK cells when compared to c-NK across all NK subgroups (P = 0.00236, P = 0.0235, P = 0.0070 respectively). Taken together, following pre-activation with IL-12, IL-15, and IL-18, there was a reduction in KIR expression in the CD56^dim^ subset, as well as an augmentation of NKp30 expression in the CD56^dim^CD16^dim^ subset and an increase in NKG2A expression in all NK populations.

### Activation mark of CIML NK cells

**Fig. 2:**
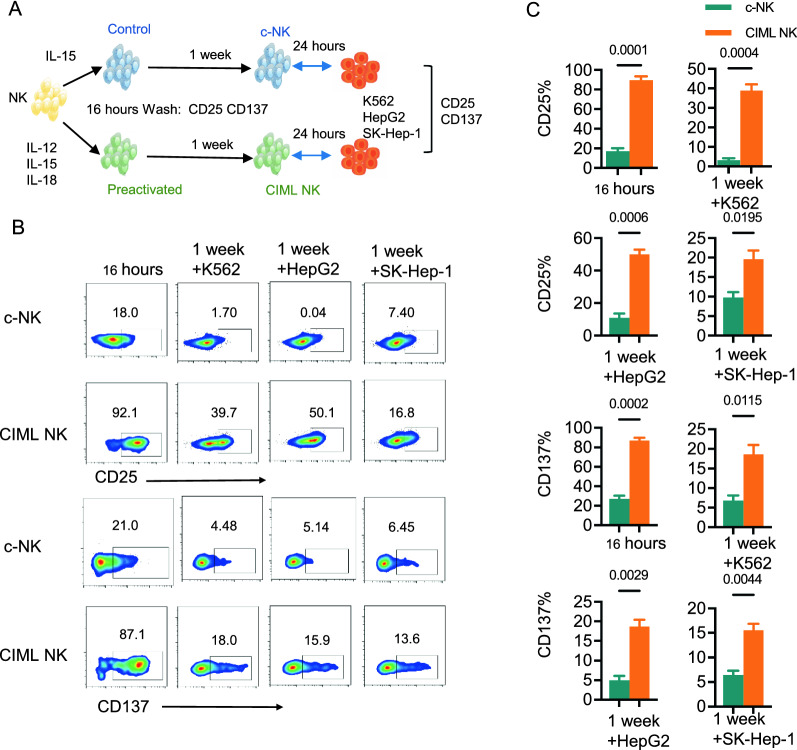
Preactivation with IL-12, IL-15, and IL-18 induces CD25 and CD137 up-regulation on CIML NK. **A** Schema of in vitro experiments. Purified NK cells were activated with IL-12, IL-15, and IL-18 or control-treated for 16 h and washed. After differentiating for 6 days, NK cell phenotype and functionality were assessed. **B** Representative flow plots showing the expression of CD25 and CD137 on NK cells after stimulation with the IL-12, IL-15, and IL-18 or low concentrations of IL-15 for 16 h and cocultured with tumor targets (K562, HepG2, and SK-Hep-1 cells) on day 7 performed at an effector-to-target ratio (E: T) of 2:1 for 24 h. Insert values are the percent positive of indicated markers for c-NK cells and CIML NK cells. **C** Graphs show the quantification of FACS analysis data. Data are presented as means ± s.e.m. Statistical significance was calculated by unpaired two-sided t-test

It has been observed that the upregulation of CD25 in NK cells results in the formation of a trimeric high-affinity receptor (CD25/122/132) that increases IL-2 signaling [21, 22]. In addition, CD137 expression is transiently seen on activated T cells, activated NK cells, and mature dendritic cells. Furthermore, CD137 activation manages the level of cytokine-driven NK cell proliferation and the NK cell life span during the culture period [23–25]. We employed CD25 and CD137 to determine if CIML NK cells can initiate an immunological response to target cells upon recall. To do so, we followed an experimental strategy: first, we measured CD25 and CD137 expression on NK cells after brief stimulation with cytokines, and then we activated NK cells with tumor cells after a 6-day rest (Fig. 2A). Flow cytometry plots demonstrated that, after IL-12, IL-15, and IL-18 stimulation of 16 h, CIML NK cells exhibited a greater expression of CD25 and CD137 when compared to c-NK Cells (Fig. 2B), with statistical significance (Fig. 2C; P = 0.0001, P = 0.0002, respectively). As well as after NK cell differentiation (on day 7) co-cultured with K562, HepG2 and SK-Hep-1 cells for 24 h at a ratio of 2:1, we also confirmed the increase in CD25 (Fig. 2C; P = 0.0004, P = 0.0006, P = 0.0195, respectively) and CD137 (Fig. 2C; P = 0.0115, P = 0.0029, P = 0.0044, respectively). It is evident that the combination of IL-12, IL-15, and IL-18 has a prompt effect on NK cells by increasing the expression of CD25 and CD137 compared to c-NK cells. Moreover, the expression of CD25 and CD137 on CIML NK cells can be restored after 6 days of rest following their cultivation with tumor targets.

### CIML NK Cells exhibit potent anti-tumor activity in vitro

**Fig. 3:**
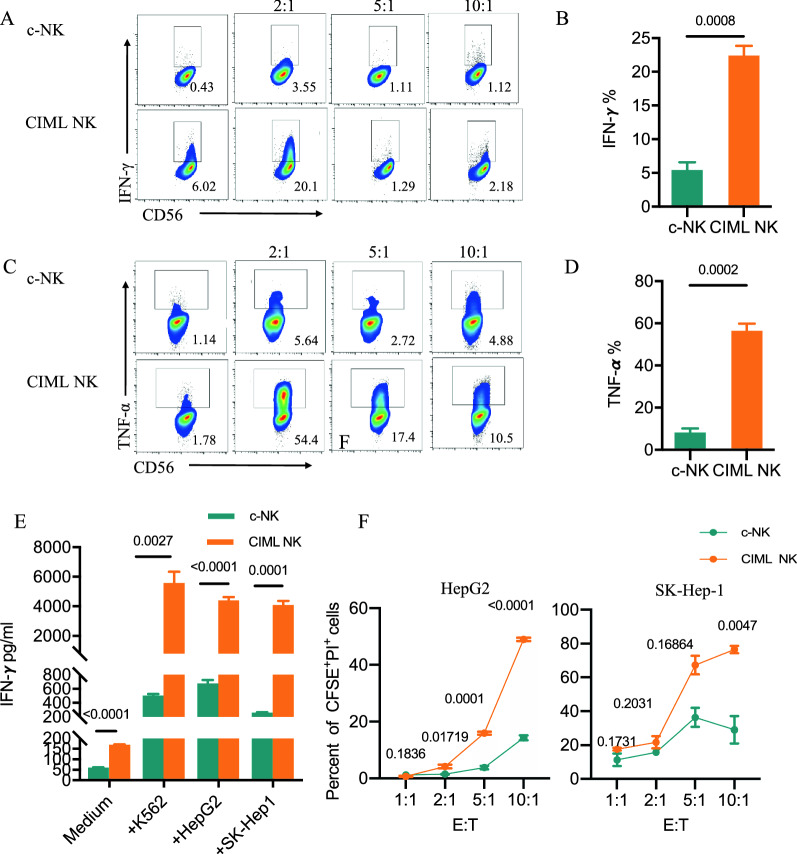
CIML NK cells exhibit enhanced functional responses against hepatocarcinoma targets. Representative bivariate mass cytometry plots of IFN-γ (**A**) and TNF-α (**C**) expressed by c-NK cells and CIML NK cells stimulated by HepG2 cells at the designated effector-to-target ratios (E: T). Summary of data (mean ± s.e.m.) from the same donor showing percentages of IFN-γ-positive NK cells (**B**) and TNF-α–positive NK cells (**D**) at an E: T of 2:1. **E** The bar graphs show a comparison of IFN-γ secretion of c-NK cells and CIML NK cells following incubation with the indicated cell lines for six hours in which K562 cells worked as a positive control group. **F** The cytotoxic reactivity of c-NK and CIML NK cells was measured using CFSE/PI cytotoxicity assay, the target cells were HepG2 (left) and SK-hep-1(right), respectively. Statistical significance was calculated by an unpaired two-sided t-test. *TNF-α* tumor necrosis factor-alpha, *IFN-γ* interferon-gamma

Flow cytometry analysis was conducted to assess the expression of activating, inhibitory, and other receptors responsible for cytotoxicity, with the aim of determining their lytic activity against HCC cell lines. The intracellular level of IFN-γ and TNF-α in CIML NK cells was then measured on day 7 by co-culturing them with HepG2 cells at various effector-to-target (E: T) ratios, including 2:1, 5:1, and 10:1 for 8 h (Fig. 3A, C). The results of stimulation indicated that the pre-activation of IL-12, IL-15, and IL-18 could significantly enhance IFN-γ and TNF-α expression levels in CIML NK cells compared to the control group. At a ratio of 2:1, CIML NK cells demonstrated a significantly higher expression of IFN-γ (P = 0.0008) and TFN-α (P = 0.0002) when compared to c-NK cells (Fig. 3B, D). Furthermore, NK cells primed with IL-12, IL-15, and IL-18 secreted higher levels of IFN-γ when restimulated with the target cells K562, HepG2, and SK-Hep-1 (P < 0.0001, all groups) (Fig. 3E). A cytotoxicity assay involving CFSE-labeled HepG2 or SK-Hep-1 cells and NK cells was conducted for a duration of 6 h. The results showed that CIML NK cells had a stronger lytic effect than c-NK cells at each E:T ratio (Fig. 3F). In accordance with augmented cytotoxicity, NK cells primed with IL-12, IL-15, and IL-18 show increased levels of granzyme B and CD107a when restimulated with the target cells HepG2 or SK-Hep-1 (Additional file 1: Fig. S1A–D). Altogether, these findings suggest increased production of cytotoxic molecules and enhanced cytotoxicity in CIML NK cells.

### CIML NK Cells modified with iRGD possess superior penetration capacity in MCSs

**Fig. 4:**
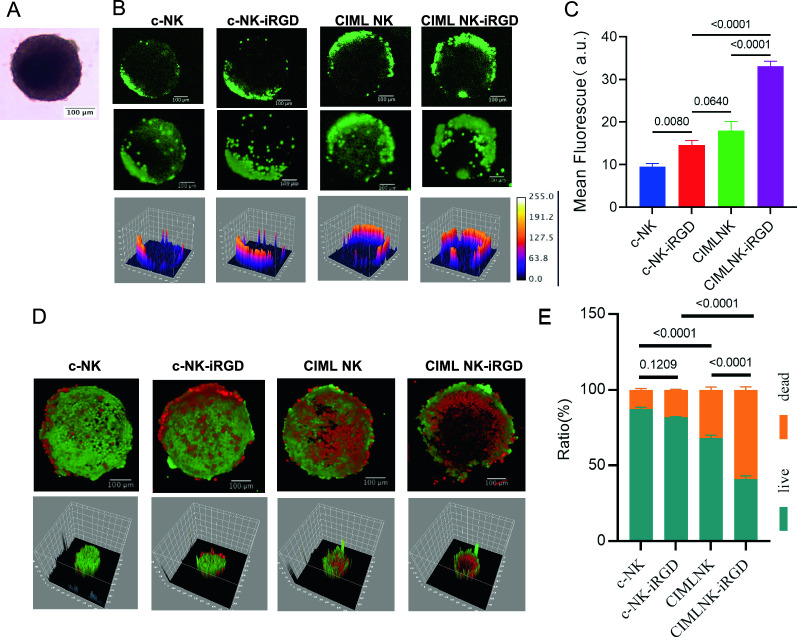
CIML NK-iRGD showed improved tumor infiltration capacity and antitumor efficiency in 3D tumor spheroids. **A** The constructed MCSs of iRGD-receptors-positive gastric cancer cell line HGC27. The image was obtained under 4 × magnification. The scale bar was 100 μm. **B** Representative morphological assessment of HGC27-MCSs was exposed to indicated CFSE stained NK cells at an effector to target cell ratio (E: T) of 5:1 calculated on the initial number of spheroids inoculated for 6 h before confocal microscopy. **C** Summary of data showing the infiltrated depth of HGC27-MCSs by indicated NK cells for 6 h by quantitative analysis of mean fluorescence intensity. Statistical significance was calculated by one-way analysis of variance (ANOVA). **D** Representative morphological assessment of HGC27-MCSs was destroyed by indicated NK cells at an E: T of 5:1 calculated on the initial number of spheroids inoculated for 6 h before Viability/Cytotoxicity Assay Kit staining and imaged by confocal microscopy. Viable HGC27 cells are stained with Calcein AM (green). Dead HGC27 cells are stained with ethidium homodimer III (EthD-III; red). **E** Summary of data from **D** showing Live/dead cell quantification in MCSs. Data are presented as means ± s.e.m. Statistical significance was calculated by unpaired two-sided t-test

Our team has found that the alteration of effector T cells with iRGD is an effective way to enhance adoptive immune cells penetrating into angiogenic vessels and tumor tissues [17, 26]. Three-dimensional (3D) cell culture systems have gained increasing interest in drug discovery and tissue engineering due to their evident advantages in providing more physiologically relevant information and more predictive data for in vivo tests [27]. HGC27 gastric cancer cell line was used to construct 3D multicellular spheres (MCSs) as detailed in a previous study (Fig. 4A). Our study aimed to assess if iRGD could improve the infiltration of CIML NK cells in MCSs. The results showed that c-NK cells alone were not able to penetrate the MCSs, with only a weak signal detected at the edges. c-NK-iRGD and CIML NK slightly improved NK penetration. In comparison, CIML NK-iRGD cells were more successful in entering round spheroids than other groups, and the fluorescence intensity was consistently increased in CIML NK-iRGD group (Fig. 4B, C). This in vitro spheroid model derived from HGC27 cell lines demonstrates that the targeting effect of iRGD is not only applicable to activated T cells, but also to NK cells.

Purified NK cells were activated with IL-12, IL-15, and IL-18 or control-treated for 16 h and washed, and then differentiated for 6 days. Initially, the CFSE/PI in vitro assay was employed to evaluate the cytotoxic potential of CIML NK and c-NK against the HGC27 gastric cancer cell line. It was found that CIML NK had a more potent cytotoxic effect than c-NK (Additional file 2: Fig. S2A, B). Afterwards, the killing capacity of iRGD-modified CIML NK was examined in HGC27 MCSs (Fig. 4D, 4E). Results indicated that c-NK and c-NK-iRGD caused minimal cell death, whereas CIML NK caused moderate cytotoxicity. Moreover, CIML NK modified with iRGD was found to be more effective in killing cells than CIML NK. (p < 0.0001). A cavity was observed in the CIML NK-iRGD group, which suggested that iRGD-endowed CIML NK cells had superior tumor infiltration and tumor-killing abilities. These results provided initial evidence that iRGD modification promoted CIML NK infiltration, a prerequisite for antitumor efficacy in vivo.

### CIML NK cells modification with iRGD potently inhibited tumor growth in HCC xenograft mouse model

**Fig. 5:**
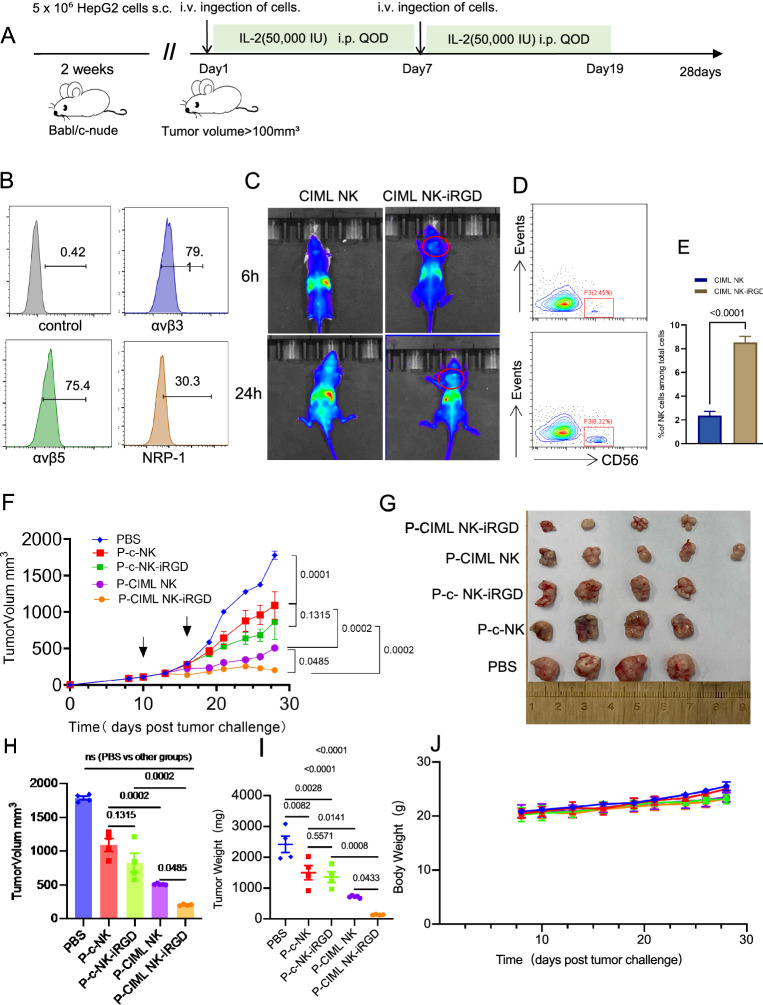
iRGD-modified P-CIML NK cells effectively controlled tumor growth. **A** Experimental scheme of the antitumor experiment in vivo. Five-week-old female Babl/c-nude mice were subcutaneously injected with 5 × 10^6^ HepG2 cells to initiate an antitumor experiment in vivo. After two weeks, 2 × 10^7^ P-CIML NK (PBMCs were primed with IL-12, IL-15, and IL-18) cells or P–c-NK (PBMCs primed with low concentration IL-15) modified with iRGD or not were injected into the mice. This was followed by IL-2 treatment every other day for approximately 10 times, with 4 or 5 mice in each treatment group. The tumor burden of the mice was monitored. **B** Histograms showing the expression of αvβ3, αvβ5 and NRP-1 on HepG2 cells. **C** Imaging of mice with subcutaneous HepG2 tumors was conducted in vivo at various intervals post intravenous injection of CIML NK (sorted CD56 + cells) cells, which had been modified with or without iRGD. Magenta dashed lines indicate tumors. CIML NK cells labeled with DiR. **D** The percentage of human CD56 + cells in the tumor tissue of CIML NK-iRGD or control CIML NK-treated mice detected by flow cytometry at 24h after intravenous injection of NK cells. **E** Summary of data from **D** showing CD56 + NK cells among total cells. **F** The tumor volume of the mice treated with P–c-NK or P-CIML NK modified with or without iRGD. **G** Photos of tumors harvested from mice in all groups on day 28 after tumor inoculation. The average tumor volume (**H**) and tumor tissue weight (**I**) in different groups at the endpoint of the animal experiment. **J** The average weight of different groups for 28 days. Statistical significance was calculated by one-way analysis of variance (ANOVA) represented as mean ± s.e.m. *NRP-1* Neuropilin-1, *s.c.* subcutaneous injection, *i.v.* intravenous injection, *i.p.* intraperitoneal

In order to assess if CIML NK cells modified with iRGD could control tumor growth in a preclinical in vivo model of HCC, an adoptive transfer was performed in a xenogeneic mouse model of HCC (Fig. 5A). This was done using HepG2 subcutaneous tumors in Babl/c-nude mice, after confirming the presence of receptors on HepG2 cells that specifically bind to iRGD, including αv integrins (αvβ3, αvβ5) and neuropilin-1 (NRP-1) receptors (Fig. 5B). We first evaluated the tumor-specific homing and extravasation of systemically delivered CIML NK-iRGD in a HCC subcutaneous tumor model. Whole-body fluorescence imaging demonstrated that, as expected, CIML NK cells alone distributed primarily in the liver, with very weak signal detected in the tumor area. iRGD modified CIML NK exhibited strong signal in tumors 24 h after cell transfusion (Fig. 5C). We created a single-cell suspension of tumors that had been treated with CIML NK or CIML NK-iRGD and evaluated the infiltration of adoptive transferred NK cells by flow cytometry. Notably, a higher proportion of CD56^+^ NK cells was observed in the iRGD-modified group (Fig. 5D, E).

Our results show that IL-12, IL-15, and IL-18 are the main activators of NK cells in the PBMCs after 16 h. After differentiated for 6 days, P-CIML-NK (PBMCs were primed with IL-12, IL-15, and IL-18) and P–c-NK (PBMCs were primed with low concentration IL-15) cocultured with tumor targets for 6 h and then detected by flow cytometer. The data showed that CD56^+^ NK cells primarily expressed CD107a, IFN-γ, and TNF-α, whereas CD3^+^ T cells had very low levels of them either in the IL-12, IL-15, and IL-18 primed PBMC or the control conditions (Additional file 3: Fig. S3A–C). On day 7, CD56^+^ and CD3^+^ NK cells were isolated from PBMCs that were either activated with IL-12, IL-15, and IL-18 or control conditions and the cytotoxicity of the four groups of cells against HCC was verified when the quantity of cells was equal. Results from the in vitro killing experiment indicated that CIML NK had the strongest killing effect with the increase of effector target ratio, whereas c-T and CIML T had very low cytotoxicity (Additional file 3: Fig. S3D). It is hypothesized that, in the absence of TCR receptor agonist and only IL-12/15/18, short-term stimulation mainly activated NK cells in PBMCs. In this study, instead of the conventional protocol for enriching pure NK cells, IL-12, IL-15, and IL-18 primed NK cells in PBMCs (P-CIML NK cells) were utilized, which was based on in vitro findings. It was estimated that the proportion of NK cells in PBMCs was approximately 30% (data not shown).

Following blow, we performed a tumor suppression test in vivo. The study demonstrated that P-CIML NK cell-based treatments were successful in suppressing tumor growth, with P-CIML NK cells modified with iRGD exhibiting the most marked difference compared to the P-CIML NK cell group (Fig. 5F, P = 0.0485). This was further evidenced by the reduced weight and volume of the growing tumor mass harvested from the P-CIML NK cell modified with iRGD group (Fig. 5G–I). Consequently, it was concluded that iRGD-modified IL-12, IL-15, and IL-18 primed PBMCs, which is mainly the CIML NK cells inside that had a higher cytotoxic potential in the xenograft model of HCC.

Clinical evaluation showed that adoptive memory NK cell therapy was safe for hematologic malignancies [28, 29]. We examined if normal target cells, for instance DC (dendritic cell), monocyte, or endothelial cells expressed iRGD receptors. Our research revealed that CD11C^+^ DC, CD14^+^ monocyte, and HUVEC (Human Umbilical Vein Endothelial Cells) did not exhibit αvβ3 or αvβ5 expression (Additional file 4: Fig. S4A). CIML NK cells did not display any cytotoxic effects when co-cultured with HUVEC cells, regardless of the effect-target ratio (Additional file 4: Fig. S4B). It is now well established that NK cells are able to discriminate between mature and immature DCs by killing the latter because of their low amount of surface human leukocyte antigen (HLA) class I molecules[30]. Then we verified the killing effect of CIML NK on mature and immature DC in vitro. The results show that immature DCs are susceptible to CIML NK cell mediated cytolysis while mature DCs are protected, which is similar to previous research by other teams (Additional file 4: Fig. S4C). Upon adoption of retransfusion of CIML, contact with immature DC may prove to be lethal for this group of cells; conversely, this may be advantageous for antitumor therapy.

In order to assess the biochemical toxicity of P-CIML NK cells modified with iRGD, a HCC xenograft mouse model was utilized. Results indicated that there was no significant difference in the body weight of the mice across the adoptive transfer groups (Fig. 5J). In addition, H&E-stained images of the main organs (heart, liver, spleen, lung, kidney) taken out after the last treatment showed no apparent damage in all groups (Additional file 5: Fig. S5A). Furthermore, hematological examinations, including serum biochemistry assays, revealed no significant differences in the serum biochemistry indexes of the mice in each group, all of which were within the normal range (Additional file 5: Fig. S5B). Consequently, it was concluded that iRGD-modified memory-like NK cells had a higher cytotoxic potential in the xenograft model of HCC.

## Discussion

Hepatocellular carcinoma (HCC) is a major health concern with an increasing prevalence and poor prognosis [31]. NK cells have been identified as potential targets for immunotherapeutic approaches in HCC treatment due to their anti-tumor activity. Studies have reported that the number and function of NK cells are significantly reduced in HCC patients, and this reduction of tumor-infiltrating NK cells is linked to poor survival in the advanced stages of HCC [32]. This highlights the importance of intrahepatic NK cells in the immune response against HCC. Consequently, various strategies have been developed to improve NK cell function and restore NK cell activity in the fight against HCC. NK cell-based adoptive transfer therapy requires expanding NK cells ex vivo, long persistence in vivo, maximal in vivo activity, and NK cell killing specificity.

Numerous studies have highlighted the powerful antitumor effects and therapeutic potential of cytokine-induced memory-like NK cells [9, 33–35]. pre-clinical studies have documented clinical responses in over half of patients with relapsed refractory acute myeloid leukemia [9], without any apparent toxicity. Moreover, the transferred NK cells proliferated, expanded, and sustained enhanced antileukemia responses in patients [8, 28]. Nevertheless, clinical studies on CIML NK cell-based immunotherapy for solid tumors are rare. The treatment of solid tumors by NK cells faces many difficulties due to tumor microenvironment (TME) and inhibitory immunity in solid tumors, leading to weak NK cell function, poor tumor trafficking, and infiltration of NK cells into the tumor. NK cells have been observed to infiltrate primary tumors of solid cancer, as well as metastases and tumor-infiltrating lymph nodes. However, most solid tumors usually have lower NK cell infiltration [36], and most studies report a decline in NK cell infiltration in malignant tissues compared to corresponding non-malignant tissues [37, 38].

We compared NK cells pre-exposed to IL-12, IL-15, and IL-18 with controls from the same donor in terms of memory-like phenotypes and effector function. Our data revealed a distinctive memory-like phenotype of CD56^dim^CD16^dim^ CIML NK cells, characterized by lower levels of KIR and higher expression of NKp30 and NKG2A. Furthermore, IL-12, IL-15, and IL-18 induced CD25 and CD137 upregulation on CIML NK cells even after incubation with cancer cell lines (K562, HepG2, and SK-Hep-1 cells) after a 6-day interval. CIML NK cells were found to express TNF-α and IFN-γ at higher levels than control NK cells in the presence of HepG2 target cells. Additionally, CIML NK cells were able to produce large amounts of IFN-γ within 6 h of incubation with cancer targets, resulting in enhanced cytotoxicity against HCC cells in vitro. In accordance with augmented cytotoxicity, NK cells primed with IL-12, IL-15, and IL-18 show increased levels of granzyme B and CD107a when restimulated with the target cells HepG2 and SK-Hep-1. This was further validated by the fact that spheroids derived from HGC27 cell lines were efficiently penetrated and killed by iRGD-modified CIML NK cells. Finally, iRGD-modified IL-12, IL-15, and IL-18 primed PBMCs, which is mainly the CIML NK cells inside that had a higher cytotoxic potential in the xenograft model of HCC.

Previous studies have identified CIML NK cells by their increased expression of CD56, NKG2A, and NKp30, as well as a modest decrease in the median expression of CD16 [9, 33]. Upon comparison of the phenotypes of CIML NK cells with sorted CD56^bright^CD16^−^, CD56^dim^CD16^+^, and CD56^dim^CD16^dim^ subsets, a modest decrease in the percentage of cells expressing KIR were observed. In our experimental system, CIML NK cells contained a stable percentage of NKp30^+^ NK cells (approximately 90%). Additionally, a significantly higher expression of NKG2A^+^ NK cells was noted in CIML NK cells compared to controls from the same donor. Our data suggest that pre-activation appears to reduce the percentage of KIR^+^CD56^dim^CD16^dim^ NK cells and enrich NKp30^+^NKG2A^+^CD56^dim^CD16^dim^ NK cells, which may be due to activation-induced shedding of CD16. Functional analysis of CIML NK cells demonstrated a much higher expression of cytokines and cytotoxicity when exposed to HCC cancer cells. Our results indicate that these memory-like NK cells are resting and have the capacity to be readily boosted, with effector memory-like NK cells being a major source of IFN-γ in the recall immune response, developing an immediate IFN-γ response.

The combination of CIML NK cells and iRGD may overcome intrinsic infiltration resistance in tumor tissues and facilitate NK cell-mediated cytotoxicity. Our research indicates that CLML NK cells modified with iRGD demonstrate greater accumulation and infiltration in the MCSs or subcutaneous hepatocellular carcinoma tumor model. Enhanced penetration, coupled with its memory characteristics, eventually translated into potent antitumor efficacy of CIML NK cells modified with iRGD, as demonstrated in xenograft mouse models. The modification of IL-12, IL-15, and IL-18 primed PBMCs with iRGD significantly reduced the growth of HepG2-cell bulk with ensured safety. However, to ensure safety, much effort is still required to verify the durability of CIML NK cells and to assess the efficacy of iRGD-modified CIML NK cells when administered through adoptive transfer. We have already conducted a clinical trial on the combination of activated T cells with iRGD-based adoptive immunotherapy (ChiCTR2200061306). Following this, we plan to initiate clinical trials of adoptive immunotherapy using iRGD-modified CIML NK cells.

Our experiment demonstrated that IL-12, IL-15, and IL-18 mainly activate NK cells, not T cells; however, our in vivo experiment utilized adoptive transfer of IL-12, IL-15, and IL-18-activated PBMCs, which has certain restrictions. To address this, we plan to use K562 cells that have been altered with CD137L to boost the CIML NK from PBMCs, thus guaranteeing that more than 90% of CD56^+^ CIML NK is transfused back in vivo.

Based on current clinical results, the potential of CIML NK cells as effective anti-tumor agents has been demonstrated [28, 29, 35, 39]. However, these cells face the challenge of tumor-associated immune suppression, including the production of immunosuppressive molecules, low nutrient levels, and hypoxic conditions, as well as the presence of regulatory T cells (Tregs) and myeloid-derived suppressor cells (MDSCs). Several strategies have been proposed to combine CIML NK cells with immunotherapeutic monoclonal antibodies targeting checkpoint inhibitory receptors to address this. For instance, a combination therapy of CTLA-4 inhibitor (Ipilimumab), CIML NK cell infusion, and interleukin-15 superagonist (N-803) is currently being evaluated for the treatment of advanced head and neck cancer (Clinical trials. gov # NCT04290546). This regimen is based on the immunomodulatory role of CTLA-4 inhibitor (ipilimumab) in depleting tumor-infiltrating Tregs, followed by the adoptive transfer of CIML NK cells. Thus, it is important to consider immunomodulatory treatments in CIML NK-iRGD cell therapy to improve the efficacy of solid tumor management.

For the first time, we examined the use of iRGD in cytokine-induced memory-like NK-based adoptive immunotherapy. Our findings showed that CIML NK cells modified with iRGD had improved penetration and killing abilities in MCSs and HCC xenograft tumor models. This combination approach could greatly improve the effectiveness of adoptive NK cell immunotherapy for various solid tumor types.

## Supplementary Information

**Additional file 1: Fig. S1.** Memory-like NK cells exhibit enhanced cytotoxicity against hepatocellular carcinoma targets. Representative flow plots showing the expression of granzyme B (A) and CD107a (B) on sorted NK after stimulation with IL-12, IL-15, and IL-18 or control condition of low-dose IL-15 for 16h and cocultured with tumor targets (HepG2 and SK-Hep-1 cells) on day 7 performed at an effector to target cell ratio (E: T) of 2:1 for 6h. (C) Summary of data from (A) showing granzyme B median fluorescence intensity (MFI). (D) Summary of data from (B) showing percentage of CD107a. Data are presented as means ± s.e.m. Statistical significance was calculated by unpaired two-sided t-test.

**Additional file 2: Fig. S2.** Memory-like NK cells exhibit enhanced cytotoxicity against HGC27 in a two-dimensional culture. Purified NK cells were activated with IL-12, IL-15, and IL-18 or control-treated for 16 hours and washed, and then differentiated for 6 days. The cytotoxic effects of CIML NK cells and control NK cells on the HGC27 gastric cancer cell line were assessed using CFSE/PI in a two-dimensional culture on day 7. The results were presented in a flow diagram (Fig. S2A) and a line graph (Fig. S2B), with each data point representing the mean ±s.e.m of the assay performed in triplicates. Statistical significance was calculated by an unpaired two-sided t-test.

**Additional file 3: Fig. S3.** IL-12, IL-15, and IL-18 are primarily responsible for activating NK cells in PBMCs and are the main source of antitumor effects in vitro. (A) Schema of in vitro experiments. IL-12, IL-15, and IL-18 primed NK cells in PBMCs (P-CIML NK) were utilized instead of the conventional protocol for enriching pure NK cells. After differentiated for one week, P-CIML-NK and P-c-NK cocultured with tumor targets at an effector to target cell ratio (E: T) of 2:1 for 6h. (B) Flow plots were used to measure the presence of CD107a, IFN-γ, and TNF-α in CD3 and CD56 positive cells. (C) The data showed that the percentage of CD107a, IFN-γ, and TNF-α on CD3^+^ T cells or CD56^+^ NK cells was significantly higher in the IL-12, IL-15, and IL-18 primed PBMC compared to the control conditions. On day 7, CD56^+^ and CD3^+^ cells were also isolated from PBMCs that were either activated with IL-12, IL-15, and IL-18 or control conditions. Following sorting, four groups of cells were obtained: CIML NK and T cells (termed CIML T) isolated from P-CIML-NK, and c-NK and T cells (termed c-T) isolated from P-c-NK. (D) The killing function of the four groups of cells on HepG2 was confirmed. Data point representing the mean ± s.e.m of the assay performed in triplicates. Statistical significance was calculated by student’s t-test Data.

**Additional file 4: Fig. S4.** Expression of αvβ3 and αvβ5 on DC, monocyte, and HUVEC cells. Integrin αvβ5 was detected using FITC-conjugated mouse anti-human αvβ5 monoclonal antibody, and integrin αvβ3 was detected using FITC-conjugated mouse anti-human αvβ3 monoclonal antibody. The matched isotype control was FITC conjugated mouse IgG1κ. (A) Expression of αvβ3 and αvβ5 on CD14+ monocyte, CD11c+ DC, and HUVEC cells. CD11c+ DC cells. (B) The cytotoxic effects of CIML NK on HUVEC cells. (C) The cytotoxic effects of CIML NK on immature DCs and mature DCs respectively.

**Additional file 5: Fig. S5.** Hematological examinations and H&E staining of major organs and in in a xenogeneic mouse model of HCC. (A)Hematological examinations: blood in all groups were harvested and tested at 28 days post-tumor implantation. (B) H&E staining: organs in all groups were harvested and stained with H&E at 28 days post-tumor implantation.

## Data Availability

The original contributions presented in the study are included in the article/additional file.

## Abbreviations

CIML NK: Cytokine-induced memory-like natural killer cells
IL: Interleukin
HCC: Hepatocellular carcinoma
MCSs: Multicellular spheroids
ACT: Adoptive cell therapy
DSPE-PEG: 1,2-Distearoyl-sn-glycero-3-phospho-ethanolamine-polyethylene glycol
FACS: Flow cytometry staining buffer
CFSE: Carboxy fluorescein succinimidyl amino ester
PI: Propidium iodide
H&E: Hematoxylin and eosin
IFN-γ: Interferon-gamma
TNF-α: Tumor necrosis factor-alpha
EthD-III: Ethidium homodimer III
NRP-1: Neuropilin-1
PBMCs: Peripheral blood mononuclear cells
TME: Tumor microenvironment
Tregs: Regulatory T cells
MDSCs: Myeloid-derived suppressor cells
