# Correction: iRGD-modified memory-like NK cells exhibit potent responses to hepatocellular carcinoma

**DOI:** 10.1186/s12967-023-04162-y

**Published:** 2023-05-08

**Authors:** Yanbing Dong, Ying Huang, Zhe Zhang, Aoxing Chen, Lin Li, Manman Tian, Jie Shen, Jie Shao

**Affiliations:** 1 Comprehensive Cancer Centre of Drum Tower Hospital, Medical School of Nanjing University, Clinical Cancer Institute of Nanjing University, Nanjing, China; 2 Comprehensive Cancer Center, Nanjing Drum Tower Hospital Clinical College of Traditional Chinese and Western Medicine, Nanjing University of Traditional Chinese Medicine, Nanjing, China; 3 Department of Oncology, The Second People’s Hospital of Huai’an, Huai’an, China

**Correction: Journal of Translational Medicine (2023) 21:205** 10.1186/s12967-023-04024-7

Following publication of the original article [1], we have been notified that one of the authors’ names was misspelled. It should be as follows:

Ying Huang^2,3†^
