# Supplementary material for: iRGD-modified memory-like NK cells exhibit potent responses to hepatocellular carcinoma
Source: J Transl Med. 2023 Mar 17;21:205. doi: 10.1186/s12967-023-04024-7 (PMC10022190; doi:10.1186/s12967-023-04024-7)
Supplement: Supplementary file 1 — Additional file 1: Fig. S1. Memory-like NK cells exhibit enhanced cytotoxicity against hepatocellular carcinoma targets. Representative flow plots showing the expression of granzyme B (A) and CD107a (B) on sorted NK after stimulation with IL-12, IL-15, and IL-18 or control condition of low-dose IL-15 for 16h and cocultured with tumor targets (HepG2 and SK-Hep-1 cells) on day 7 performed at an effector to target cell ratio (E: T) of 2:1 for 6h. (C) Summary of data from (A) showing granzyme B median fluorescence intensity (MFI). (D) Summary of data from (B) showing percentage of CD107a. Data are presented as means ± s.e.m. Statistical significance was calculated by unpaired two-sided t-test. [file 12967_2023_4024_MOESM1_ESM.pptx]

## Slide 1
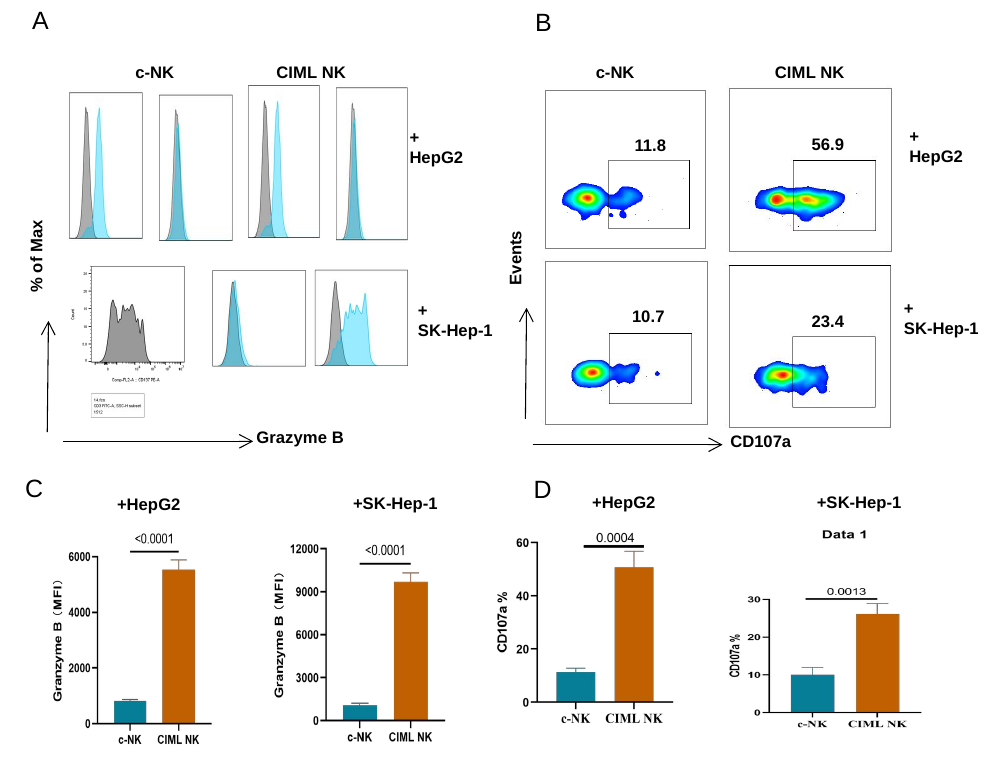

A
B
| c-NK | CIML NK | c-NK | CIML NK |
| --- | --- | --- | --- |
% of Max
+
HepG2
+
HepG2
56.9
11.8
Events
+
SK-Hep-1
+
SK-Hep-1
10.7
23.4
Grazyme B
CD107a
C
D
+SK-Hep-1
+HepG2
+SK-Hep-1
+HepG2
