# Supplementary figures and images for: iRGD-modified memory-like NK cells exhibit potent responses to hepatocellular carcinoma
Source: J Transl Med. 2023 Mar 17;21:205. doi: 10.1186/s12967-023-04024-7 (PMC10022190; doi:10.1186/s12967-023-04024-7)

## Slide 1
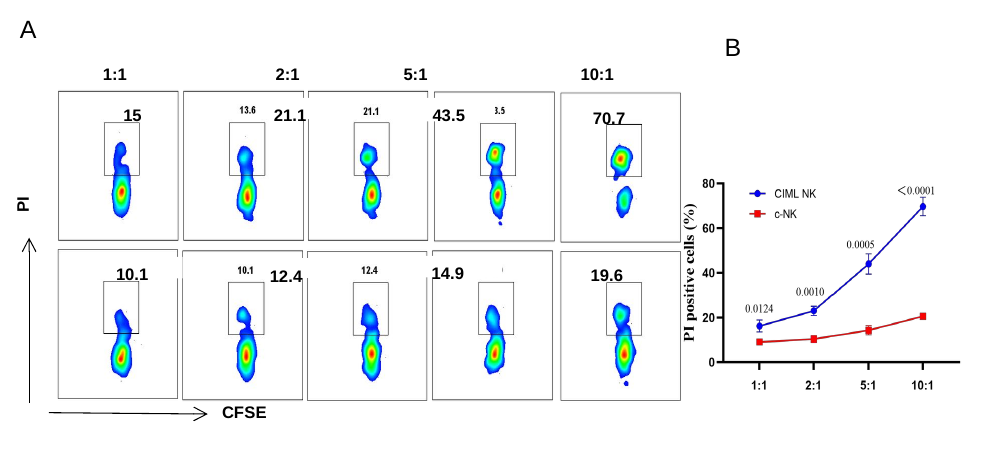

A
B
| 1:1 | 2:1 | 5:1 | 10:1 | |
| --- | --- | --- | --- | --- |
15
21.1
43.5
70.7
14.9
10.1
19.6
12.4
PI
CFSE

Supplement: Supplementary file 2 — Additional file 2: Fig. S2. Memory-like NK cells exhibit enhanced cytotoxicity against HGC27 in a two-dimensional culture. Purified NK cells were activated with IL-12, IL-15, and IL-18 or control-treated for 16 hours and washed, and then differentiated for 6 days. The cytotoxic effects of CIML NK cells and control NK cells on the HGC27 gastric cancer cell line were assessed using CFSE/PI in a two-dimensional culture on day 7. The results were presented in a flow diagram (Fig. S2A) and a line graph (Fig. S2B), with each data point representing the mean ±s.e.m of the assay performed in triplicates. Statistical significance was calculated by an unpaired two-sided t-test. [file 12967_2023_4024_MOESM2_ESM.pptx]

## Slide 1
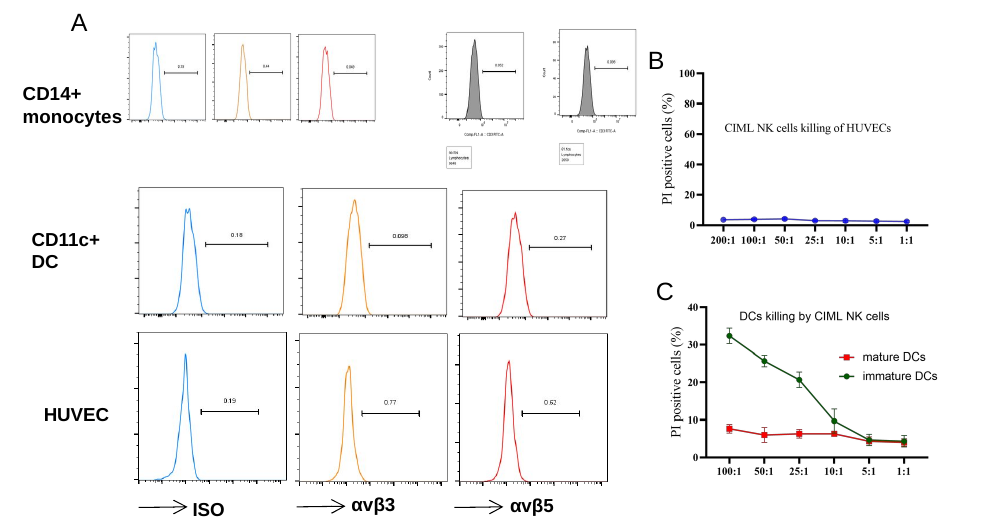

A
B
CD14+
monocytes
CD11c+
DC
C
HUVEC
 αvβ3
 αvβ5
ISO

Supplement: Supplementary file 4 — Additional file 4: Fig. S4. Expression of αvβ3 and αvβ5 on DC, monocyte, and HUVEC cells. Integrin αvβ5 was detected using FITC-conjugated mouse anti-human αvβ5 monoclonal antibody, and integrin αvβ3 was detected using FITC-conjugated mouse anti-human αvβ3 monoclonal antibody. The matched isotype control was FITC conjugated mouse IgG1κ. (A) Expression of αvβ3 and αvβ5 on CD14+ monocyte, CD11c+ DC, and HUVEC cells. CD11c+ DC cells. (B) The cytotoxic effects of CIML NK on HUVEC cells. (C) The cytotoxic effects of CIML NK on immature DCs and mature DCs respectively. [file 12967_2023_4024_MOESM4_ESM.pptx]

## Slide 1
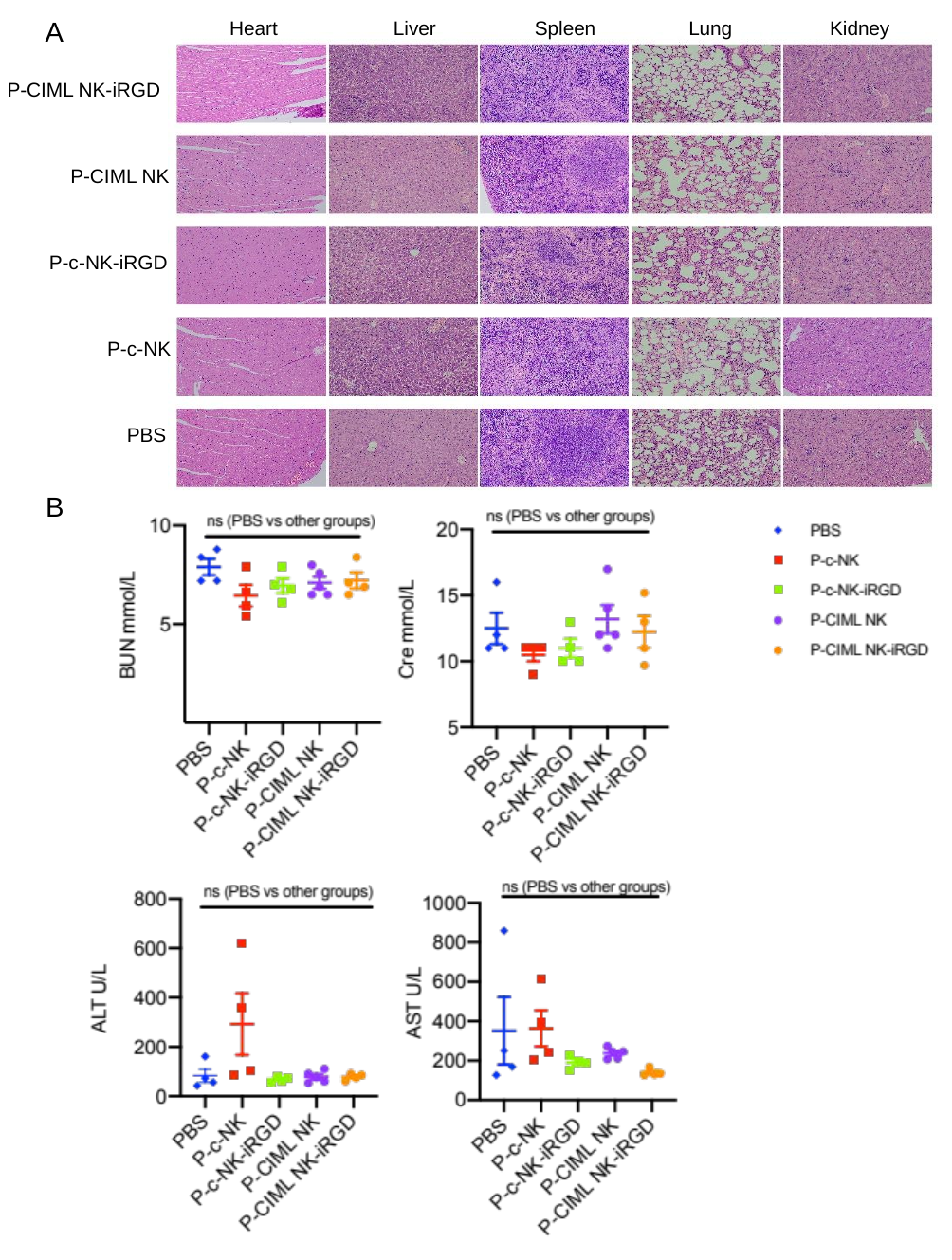

A
Liver
Spleen
Lung
Heart
Kidney
P-CIML NK-iRGD
P-CIML NK
P-c-NK-iRGD
P-c-NK
PBS
B

Supplement: Supplementary file 5 — Additional file 5: Fig. S5. Hematological examinations and H&E staining of major organs and in in a xenogeneic mouse model of HCC. (A)Hematological examinations: blood in all groups were harvested and tested at 28 days post-tumor implantation. (B) H&E staining: organs in all groups were harvested and stained with H&E at 28 days post-tumor implantation. [file 12967_2023_4024_MOESM5_ESM.pptx]
