# Supplementary material for: iRGD-modified memory-like NK cells exhibit potent responses to hepatocellular carcinoma
Source: J Transl Med. 2023 Mar 17;21:205. doi: 10.1186/s12967-023-04024-7 (PMC10022190; doi:10.1186/s12967-023-04024-7)
Supplement: Supplementary file 3 — Additional file 3: Fig. S3. IL-12, IL-15, and IL-18 are primarily responsible for activating NK cells in PBMCs and are the main source of antitumor effects in vitro. (A) Schema of in vitro experiments. IL-12, IL-15, and IL-18 primed NK cells in PBMCs (P-CIML NK) were utilized instead of the conventional protocol for enriching pure NK cells. After differentiated for one week, P-CIML-NK and P-c-NK cocultured with tumor targets at an effector to target cell ratio (E: T) of 2:1 for 6h. (B) Flow plots were used to measure the presence of CD107a, IFN-γ, and TNF-α in CD3 and CD56 positive cells. (C) The data showed that the percentage of CD107a, IFN-γ, and TNF-α on CD3+ T cells or CD56+ NK cells was significantly higher in the IL-12, IL-15, and IL-18 primed PBMC compared to the control conditions. On day 7, CD56+ and CD3+ cells were also isolated from PBMCs that were either activated with IL-12, IL-15, and IL-18 or control conditions. Following sorting, four groups of cells were obtained: CIML NK and T cells (termed CIML T) isolated from P-CIML-NK, and c-NK and T cells (termed c-T) isolated from P-c-NK. (D) The killing function of the four groups of cells on HepG2 was confirmed. Data point representing the mean ± s.e.m of the assay performed in triplicates. Statistical significance was calculated by student’s t-test Data. [file 12967_2023_4024_MOESM3_ESM.pptx]

## Slide 1
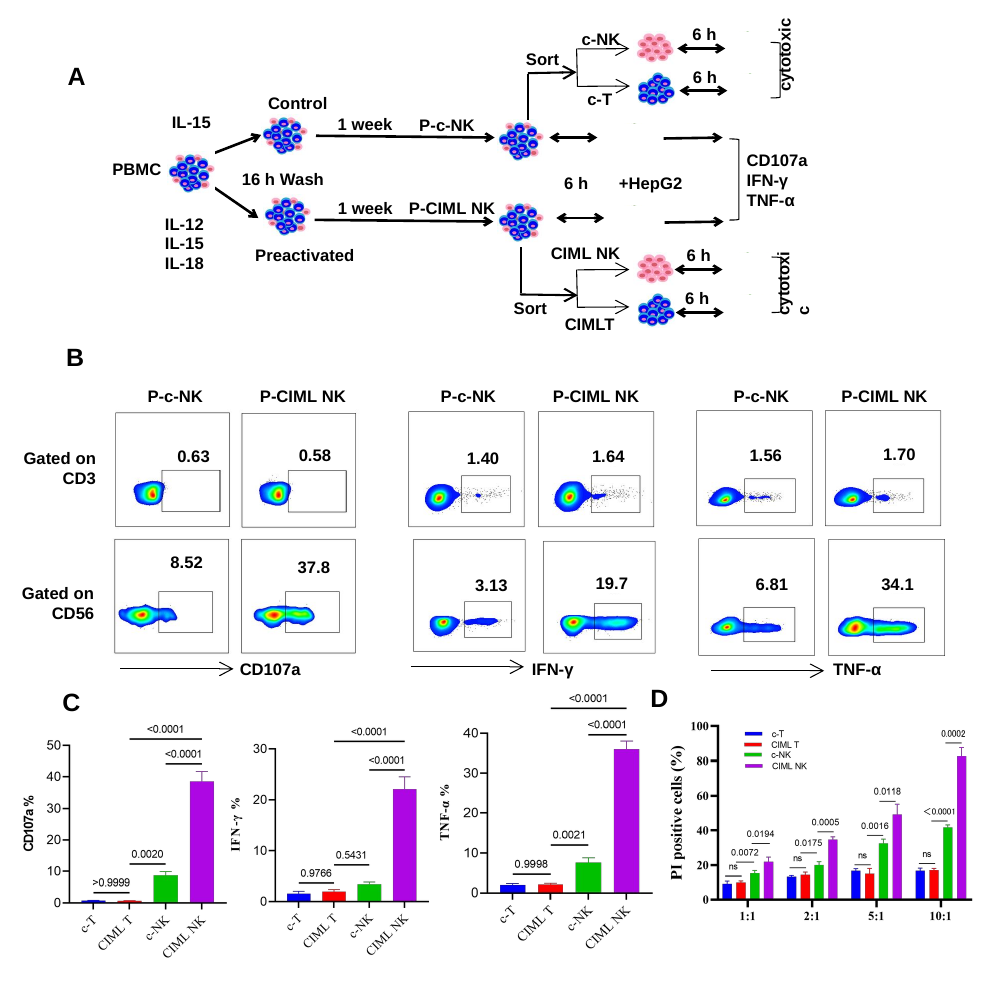

cytotoxic
6 h
c-NK
Sort
A
6 h
c-T
Control
IL-15
1 week
P-c-NK
CD107a
IFN-γ
TNF-α
PBMC
16 h Wash
6 h
+HepG2
P-CIML NK
1 week
IL-12
IL-15
IL-18
cytotoxic
CIML NK
Preactivated
6 h
6 h
Sort
CIMLT
B
P-c-NK P-CIML NK P-c-NK P-CIML NK P-c-NK P-CIML NK
Gated on CD3
1.70
0.58
1.56
0.63
1.64
1.40
8.52
37.8
19.7
34.1
6.81
3.13
Gated on CD56
CD107a
IFN-γ
TNF-α
D
C
